# Supplementary material for: Serum metabolomic analysis of the dose-response effect of dietary choline in overweight male cats fed at maintenance energy requirements
Source: PLoS One. 2023 Jan 23;18(1):e0280734. doi: 10.1371/journal.pone.0280734 (PMC9870128; doi:10.1371/journal.pone.0280734)
Supplement: Attachment — Submitted filename: Rankovic-Response to Reviewers.pdf [file pone.0280734.s001.pdf]

UNIVERSITY of GUELPH
ONTARIO VETERINARY COLLEGE
DEPARTMENT OF BIOMEDICAL SCIENCES

Mikhail Y. Golovko, PhD
Academic Editor
PLOS ONE
1265 Battery Street, Suite 200
San Francisco, CA 94111
United States

Date: 26/11/22

Dear Dr. Golovko

Thank you for the opportunity to revise our manuscript – *Rankovic et al., Serum metabolomic analysis of the dose-response effect of dietary choline in overweight male cats fed at maintenance energy requirements* - for publication in PLOS ONE.

We appreciate the careful review and constructive suggestions, and would like to thank the editor and reviewers for their time. It is our belief that the manuscript is substantially improved after making the suggested edits. We hope these changes are satisfactory for publication in PLOS ONE.

Changes made in the manuscript are highlighted.

Thank you for considering the publication of our manuscript.

On behalf of the authors,

Alexandra Rankovic, PhD
arankovi@uoguelph.ca

Department of Biomedical Sciences
Ontario Veterinary College
University of Guelph
50 Stone Road East
Guelph, Ontario, Canada N1G 2W1
T 519-824-4120
ovc.uoguelph.ca/biomedical-sciences/

IMPROVE LIFE.

Reviewer 1

1. **Firstly, I think that the title of the manuscript should reflect that the authors did this work in male cats. Within the manuscript adult cats should be revised to male adult cats.**

Response: Thank you for this suggestion. We have made the suggested change within the title and throughout the manuscript.

2. **I think there should also be a statement made in the discussion, as well. Do the authors have any data on female cats or ideas on how the data would look in those animals?**

Response: We have further elaborated on the inclusion of only male cats in the discussion and the potential limitations associated with this.

Page 29 (Line 651): "*....the use of male cats in the present study was the result of previous work investigating choline supplementation following gonadectomy in kittens fed to mimic ad libitum feeding [48,99]. Male cats have previously been found to be at greater risk of obesity as compared to females [5,13,117,118]. Although there appear to be no differences in endogenous choline synthesis in cats based on sex and gonadectomy in cats [119], healthy female cats have been reported to have lower concentrations of plasma and hepatic TAG, but the same concentrations of circulating PC, as compared to male cats [109]. Therefore, differences in hepatic lipid mobilization with additional choline supplementation between female and male cats may exist. Although women have been found to have lower betaine-homocysteine-methyltransferase (BHMT) activity, as compared to men [120], the impact on circulating sex steroids and differences between sexes on one-carbon metabolism in cats has not been studied and may also change the lipotropic effects of choline dose in cats*."

3. **The sentence below is not clear.**
   Page 12: Choline dose did not impact 261 any of the other one-carbon metabolites, including choline, creatine, formic acid and L-carnitine 262 (P Dose = 0.874, 0.432, 0.353, and 0.116, respectively).

Response: We have made the necessary change to further clarify this sentence.

Page 12 (Line 258): "*Choline dose did not impact the other one-carbon metabolites investigated: choline, creatine, formic acid and L-carnitine (P_{Dose} = 0.874, 0.432, 0.353, and 0.116, respectively).*"

4. **Within the results section can the authors add a brief statement of why each test was conducted?**

Response: We appreciate the reviewer's suggestion. However, the reasoning and use of metabolomics and these tests was addressed at the end of the introduction.

Page 5 (Line 103): "*The application of serum metabolomics allows for a comprehensive view of complex metabolic pathways, and how these pathways are altered by dietary choline intake. By quantifying low molecular weight metabolites in serum through quantitative nuclear magnetic resonance (NMR) spectroscopy and direct flow injection mass spectrometry (DI-MS), the present study aimed to establish changes in biochemical pathways…*"

5. **The authors discuss the impact of choline supplementation increasing one-carbon metabolites. Do they think this would impact other diseases such as cancer? There have been studies investigating the role of folic acid supplementation on cancer**

Response: We thank the reviewer for taking the time to make this thoughtful suggestion.

However, other diseases such as cancer are out of the scope of the objectives of this research. Our research and the use of choline supplementation in overweight cats focused specifically on its potential benefits for hepatic health maintenance.

Reviewer 2

1. **The authors should clarify why "The one lean cat was enrolled to balance BW between groups but was not included in the statistical analysis." If it's not included in the analysis should it also not be included in the BW calculations?**

Response: Thank you for this suggestion.

The lean cat was enrolled at the start of the trial, but was not included within the statistical analysis or final results as its lean body condition did not match our objectives for this research.

We are in agreement with the reviewer that its BW should therefore not be included in the BW calculations. We have therefore chosen to omit the lean cat from the manuscript and from the BW calculations in order to avoid any confusion.

2. **In Table 1 and other tables, it would be good to show the number of cats in each group.**

Response: Thank you for this constructive comment. However, the number of cats is already included within the title of each table (ie: n=14). As this study was performed as a latin square design, all 14 cats received each of the 5 doses supplemented. For this reason, the number of cats consuming each individual supplement was not included within the tables as it would be repetitive.

3. **The authors are suggesting that choline supplementation may prove useful in the prevention of FHL. However, it is not clear if the cats are at risk for FHL besides being obese. This should be clarified a bit.**

Response: We appreciate the reviewer's suggestion, and are in agreement. We have added the following to the discussion:

Page 30 (Line 676): *"Although the cats in the present study were fed at maintenance energy requirements, increasing body condition score can result in greater hepatic TAG and greater risk of insulin resistance [49, 122]."*

4. **The authors show different metabolites changing levels in different choline concentrations, however, it is not clear in the manuscript how choline will help prevent FHL when FHL was not shown in these cats. A clarification would be helpful.**

Response: The fourth paragraph of the introduction contains information focused on clarifying this:

Page 4 (Line 85): *"Given the metabolic pathways that choline participates in, it has been proposed that choline supplementation may prove useful in the prevention of FHL [45]. However, before choline supplementation can be assessed as a possible nutritional intervention for obese cats undergoing weight loss, an adequate dose must be determined…Choline supplementation above the NRC recommendations could benefit one-carbon metabolism and fatty acid oxidation. ... it remains unclear how choline supplementation may affect one-carbon and lipid metabolism in adult cats, and those with an increased liver TAG, as seen in obese cats [49]. Additionally, the dose at which choline would most benefit both PC synthesis and one-carbon metabolism in cats has yet to be determined.* "

However, we have also added the following to the discussion in hopes of further clarifying this:

Page 30 (Line 668): *"Choline has become a nutrient of interest in the maintenance of hepatic health and FHL prevention in overweight and obese cats during energy restriction, due its roles in one-carbon metabolism, phospholipid biosynthesis, and β-oxidation through L-carnitine [75,121]."*
